# Application of Chitosan in Bone and Dental Engineering

**DOI:** 10.3390/molecules24163009

**Published:** 2019-08-19

**Authors:** Alicia Aguilar, Naimah Zein, Ezeddine Harmouch, Brahim Hafdi, Fabien Bornert, Damien Offner, François Clauss, Florence Fioretti, Olivier Huck, Nadia Benkirane-Jessel, Guoqiang Hua

**Affiliations:** 1 INSERM (French National Institute of Health and Medical Research), UMR 1260, Regenerative Nanomedicine (RNM), FMTS, 11 Rue Humann, 67000 Strasbourg, France; 2 Université de Strasbourg, Faculté de Chirurgie Dentaire de Strasbourg, 8 Rue Sainte-Elisabeth, 67000 Strasbourg, France; 3 Hôpitaux Universitaires de Strasbourg, Pôle de Médecine et de Chirurgie Bucco-Dentaires, 67000 Strasbourg, France

**Keywords:** chitosan, bone engineering, regeneration, scaffold, periodontitis, dental pulp

## Abstract

Chitosan is a deacetylated polysaccharide from chitin, the natural biopolymer primarily found in shells of marine crustaceans and fungi cell walls. Upon deacetylation, the protonation of free amino groups of the d-glucosamine residues of chitosan turns it into a polycation, which can easily interact with DNA, proteins, lipids, or negatively charged synthetic polymers. This positive-charged characteristic of chitosan not only increases its solubility, biodegradability, and biocompatibility, but also directly contributes to the muco-adhesion, hemostasis, and antimicrobial properties of chitosan. Combined with its low-cost and economic nature, chitosan has been extensively studied and widely used in biopharmaceutical and biomedical applications for several decades. In this review, we summarize the current chitosan-based applications for bone and dental engineering. Combining chitosan-based scaffolds with other nature or synthetic polymers and biomaterials induces their mechanical properties and bioactivities, as well as promoting osteogenesis. Incorporating the bioactive molecules into these biocomposite scaffolds accelerates new bone regeneration and enhances neovascularization in vivo.

## 1. Introduction

The first bioerodable artificial polymer-cell scaffold was implanted into animals 30 years ago [1]. Since then, tissue engineering has become an interdisciplinary field that applies the principles of engineering and life sciences toward the development of biological substitutes that restore, maintain, or improve tissue structure and function [2]. Several biomaterials have been used for the fabrication of the scaffolds, including natural materials derived from animals or plants (collagen, starch, gelatin, alginate, cellulose, fibrin, hyaluronan, and chitosan) and synthetic materials, such as bioactive ceramics and a wide range of synthetic polymers. However, the excellent bio-based 3-dimentional (3D) polymer scaffolds should not only be non-toxic, biocompatible, and biodegradable, but also be competent in promoting cell adhesion and retaining the metabolic functions of attached cells [3], as the 3D polymer scaffolds used in tissue engineering should mimic and provide an actual in vivo surrounding microenvironment for the incorporation of cells or growth factors to regenerate damaged tissues or organs [4]. The immunomodulatory biological effects of chitosan-based scaffold have also been described. In this regard, chitosan becomes one of the most commonly studied polymers in the scientific research, not only for biopharmaceutical and biomedical applications, but also for food science and technology [5].

## 2. Results

### 2.1. Chitosan

**Figure 1:**
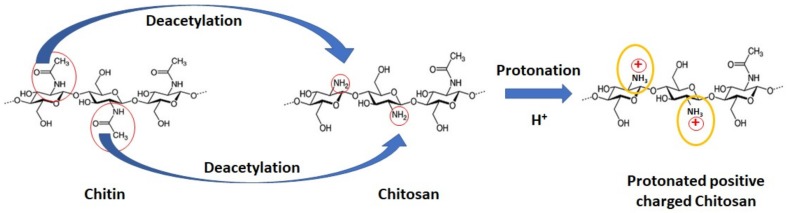
Structures for chitin, the noncharged form of chitosan, and protonated positive-charged chitosan.

Chitosan (CS) is a linear, semi-crystalline polysaccharide composed of β-(1→4)-2-acetamido-2-deoxy-b-d-glucan (*n*-acetyl d-glucosamine) and β-(1→4)-2-amino-2-deoxyb-d-glucan (d-glucosamine) units [6,7]. Its molecular weight ranges from 10 to over 1000 kDa. Chitosan is not extensively present in the environment, however, it can be easily produced via the alkaline *n*-deacetylation process of the natural biopolymer commonly found in the shells of marine crustaceans and in fungi cells walls—the chitin [8,9] (Figure 1). The deacetylation degree (DD) of chitosan, which gives an indication of the number of amino groups along the chains, is calculated as the ratio of d-glucosamine to the sum of d-glucosamine and *n*-acetyl d-glucosamine [7,10,11].

Chitin is a white, hard, inelastic, nitrogeneous polysaccharide. It is hydrophobic and is not soluble in water and most organic solvents, except for hexafluroisopropanol, hexafluroacetone, and chloroalcohols [12]. This poor solubility of chitin is an extreme limit for its practical applications. However, the free amino groups of the d-glucosamine residues of chitosan, which could be protonated, provide a better solubility for chitosan by forming a non-Newtonian, shear-thinning fluid in most diluted acidic solutions at a pH below 6.5 (pKa value ~6.3) [7,13] (Figure 1). With protonated amino groups, chitosan becomes a polycation and could subsequently form ionic complexes with a wide variety of natural or synthetic anionic species, for example, DNA, proteins, lipids, or negatively charged synthetic polymers such as poly(acrylic acid) [7,14,15].

Chitosan can be biodegraded into non-toxic residues by lysozyme or chitinase, which hydrolyses glucosamine-glucosamine, glucosamine-*n*-acetyl-glucosamine, and *n*-acetyl-glucosamine-*n*-acetyl-glucosamine linkages [16,17,18]. The rate and extent of chitosan’s biodegradability in living organisms are highly related to the molecular mass of the polymer and its deacetylation degree (DD) [19,20]. The chitosan DD can also influence its biocompatibility. A higher DD increases the number of positive charges which increases the interaction between chitosan and cells, leading to an improved biocompatibility [21]. In addition, chitosan is a low-cost and economic natural biopolymer [22]. The price of chitin (3.6–6.0 US$/kg)/chitosan (30–500 US$/kg) are hundreds or thousands of times higher than the price of shell wastes (0.05–0.15 US$/kg) [23], while the production costs were around 1.70 US$/kg for chitin and 3.50 US$/kg for chitosan 40 years ago [24].

### 2.2. Medical and Pharmaceutical Properties of Chitosan

As a natural multifunctional polysaccharide, chitosan has been widely studied for biomedical, surgical, and tissue engineering and pharmaceutical application, thanks to its biocompatibility, biodegradability, and muco-adhesiveness. Chitosan is reported to be increasingly used in the United States as an over-the-counter cholesterol-lowering agent. Positively-charged deacetylated chitosan could bind negatively charged molecules, such as fatty acids, lipids, and bile acids, in the intestinal tract, excreting these molecules from the body [25]. Chitosan is thus considered as a promising candidate for obesity and hypercholesterolemia treatment [26]. Chitosan could be also used for wastewater treatment and beverage clarification because of its good chelating or binding capacity of protonable amino groups for various species, such as metal ions [27,28]. However, the most important medical and pharmaceutical applications of chitosan are drug delivery, wound dressings, and biocomposite scaffolds for tissue engineering.

#### 2.2.1. Drug Delivery

The positively charged protonable amino group of the d-glucosamine residues of chitosan can interact with the negatively charged sialic acid residues of the glycoprotein which composes the mucus. Thus, the muco-adhesion is directly related to the DD of chitosan, with higher chitosan DD resulting in increased positive charges which enhance its muco-adhesive properties [29]. It has also been reported that chitosan can interact with the negative part of the cell membrane, enhancing the penetration of an active agent through the epithelium layer that contains tight junctions [30,31]. Consequently, owing to the muco-adhesion and enhanced penetration properties, chitosan is a suitable excipient to prepare oral, nasal, ocular, vaginal, and subcutaneous delivery forms and be used as a vaccine adjuvant or co-adjuvant to enhance the bioavailability and immunogenicity of antigens [32,33,34].

#### 2.2.2. Wound Dressings

Wound healing is a particular biological phenomenon, which progresses through a series of inter-reliant and corresponding stages to regenerate the integrity of damaged tissue and replacement of lost tissue [35]. The hemostatic and antimicrobial properties of chitosan enable its application in wound dressings [35,36,37].

Again, as a natural positive-charged polysaccharide, protonable amino groups on the chitosan backbone electrostatically interact with the various negatively charged proteins and glycolipids on the surface of red blood cells (RBC). This interaction increases blood viscosity, activates platelet adhesion and aggregation, and enhances the transportation of platelets to the vascular wall for physiological hemostasis. Blood clots are formed by intensive aggregation of RBC around the wound site to quickly stop bleeding [38,39,40,41]. Thus, the number of positive charged amino groups on chitosan directly plays an important role in its hemostatic property.

The antimicrobial activity of chitosan has been demonstrated against different microorganisms both in vivo and in vitro, such as bacteria (either Gram-positive or Gram-negative), yeast, fungi, and algae, which makes chitosan a good candidate as an antimicrobial agent in solution, film, and composite [35,42,43,44,45,46]. This antimicrobial property of chitosan has been related to the presence of its cationic nature. However, this antimicrobial activity could also depend on other intrinsic factors (for example, the type of chitosan or the degree of chitosan polymerization and deacetylation) and extrinsic factors (such as the live host, the microorganisms, and the environmental conditions). Until now, the antimicrobial activity of chitosan has not yet been fully understood.

### 2.3. Chitosan-Based Scaffold Preparation

The most common method to generate chitosan scaffolds is by freezing and lyophilizing chitosan solution. The spaces occupied by ice crystals formed in frozen chitosan solution are emptied during the sublimation, leading to the formation of pores. However, a precise control of temperature is required to form good pore structures [47,48]. Another method to form porous scaffolds is called salt leaching [49,50]. Salt crystals such as NaCl are used as porogens and put into a mold, and chitosan is then poured over the salt, penetrating into all the small spaces left between the salt crystals. The mold is then heated to melt the chitosan powder in an oven for a sufficient time. The chilled chitosan/NaCl mixture is then separated from the mold and the salt is washed away by water or alcohol, generating open pores in the chitosan scaffolds. In addition, fibrous chitosan scaffolds are formed by electrospinning. An applied electric field causes the elongation of the chitosan drop and leads to the formation of long fibers ranging from the submicron level to several nanometers in diameter [51,52].

Besides the dried chitosan-based scaffold preparation discussed above, chitosan-based scaffolds could also be prepared as hydrogels [53]. A hydrogel is a network of the same or different types of cross-linked polymer chains with good water absorption capacity. Chemically cross-linked hydrogels are formed by covalent linking of chitosan macromers, in which the bond formation is irreversible. Covalent cross-linking can also be formed between polymers and a cross-linker [54], or via photopolymerization [55]. Another way to prepare chitosan-based hydrogel is by physical cross-linking through ionic interactions. Polyelectrolyte complex networks are formed via ionic interactions between positively charged chitosan and anions or other negatively charged polymers. Although chemically cross-linked chitosan-based hydrogels show better stability and resistance to environmental variables, physically cross-linked hydrogels are more biocompatible because of the lack of chemical cross-linkers.

### 2.4. Chitosan-Based Scaffolds for Bone Regeneration

Bone is a hard and highly functionalized connective tissue constituting the skeletal framework of the human body. It supports fleshy structures, protects vital organs, and is involved in various physiological functions such as the maintenance of phosphocalcic homeostasis [56,57]. After an injury, if the impairment is mild and the defect size is small, bone tissue has certain self-healing potential through osteogenic differentiation of bone marrow mesenchymal stem cells, bone neoformation, and neo-angiogenesis at the lesion site. For large-size bone defects, bone grafts are usually needed [58,59], however, these clinical procedures have many disadvantages [60].

In the context of bone engineering, the physiological inertness and low toxic effects of chitosan-based scaffolds have been demonstrated by numerous in vitro and in vivo studies (Table 1). In addition, no allergic and inflammatory reactions upon chitosan-based material implantation, injection, or topical application in the human body have been proven [61,62,63]; however, the use of chitosan as scaffold is limited by having reduced bioactivities and mechanical properties. This disadvantage has been overcome by mixing the chitosan scaffolds with other synthetic or natural polymers [poly(vinyl alcohol), poly-ɛ-caprolactone, alginate, collagen, silk fibroin, etc.], biomaterials (hydroxyapatite, β-tricalcium phosphate, SiO_2_, etc.), or bioactive pharmacological molecules (bone morphogenetic protein 2 (BMP-2), vascular endothelial growth factor (VEGF), bisphosphonate, etc.).

Indeed, blending chitosan with most of the aforementioned polymer and/or biomaterials efficiently reinforces its mechanical properties and improves its bioactivities, such as increased protein absorption and increased biomineralization (Table 1). However, many of the published biocomposite scaffolds could not decrease the biodegradation rate of chitosan, which affects their long-term in vivo persistence (Table 1). Water retention ability is another parameter important for tissue engineering scaffolds, as the increased water retention ability of scaffold implanted in vivo could lead to the loosening and dislocation of the implant. As summarized in Table 1, most of the biocomposite scaffolds reported controls and decreased their water retention ability.

Most importantly, both in vitro and in vivo experiments demonstrated that all these chitosan-based biocomposite scaffolds are not toxic, and have very good properties of biocompatibility, osteoconductivity, and osteogenesis, promoting good cell proliferation and cell adhesion, causing an increase in new bone regeneration. Regarding more specific clinical applications of chitosan in alveolar bone and jawbone regeneration, several preclinical reports showed promising results, even for accelerating dental implant osseointegration and reconstructing critical size defects [64,65,66,67].

Over the last decade, the functionality of these chitosan-based biocomposite scaffolds have been enhanced by incorporating bioactive molecules into drug delivery systems. Therefore, bioactive molecules could be locally delivered with an adequate dose for a desired period, avoiding the drug release to non-target sites. Bone morphogenetic proteins (BMPs) are a group of growth factors originally discovered by their ability to induce bone and cartilage formation [68]. Transforming growth factor beta 1 (TGF-β1) is a polypeptide member of the transforming growth factor beta superfamily of cytokines, controlling cell growth, cell proliferation, and cell differentiation in many cell types. Studies have shown that chitosan-based biocomposite associated with either BMP2, BMP-7 or TGF-β1 efficiently induced osteogenesis and promoted a large amount new bone formation compared with control or sham groups (see Table 1). Vascular endothelial growth factor (VEGF) is a signal protein stimulating the formation of blood vessels. Incorporating VEGF into chitosan-based biocomposite scaffolds induces angiogenesis and enhances neovascularization in bone healing (see Table 1).

### 2.5. Chitosan-Based Application for Dental Engineering: The Case of Periodontal Regeneration

Periodontitis is a chronic inflammatory disease induced by bacterial infection, affecting tooth-supporting tissues and leading to significant destruction of the periodontium (i.e., gingiva, alveolar bone, periodontal ligament, cement). Such a disease is common in the global population (>50% in the U.S. population) and is the main cause of tooth loss [69]. It is characterized by the development of the periodontal pocket and, in severe cases, of infrabony defects. Treatment of such lesions represent a challenge for the clinician [70,71]. Indeed, periodontal treatment aims to reduce the inflammation and to control the infection by chemical (antibiotics, antiseptics) and mechanical treatments (scaling and root planing). However, such a treatment strategy is associated mainly to wound repair, characterized by a long junctional epithelium. In most severe cases (probing pocket depth (PPD) > 5 mm), non-surgical treatment alone could be insufficient in emphasizing the need of adjuvant therapy [72,73]. Therefore, the use of local delivery of active drugs or compounds has been suggested to target inflammation and infection, and in promoting tissue regeneration [74,75,76,77]. During the last decade, a specific interest was made towards the chitosan-based delivery system [78]. Several chitosan-based devices have been designed and evaluated in specific contexts, including in micro/nanoparticles, fibers, membrane, and gels [79,80,81]. Chitosan gels exhibit interesting physical properties that could be modulated by the concentration of chitosan. Indeed, it was shown that such chitosan-based gels (1–4%) have an interesting viscosity to be injected within periodontal pockets. Most importantly, they can be used as a reliable vehicle to release active drugs at the disease site. While a sustained release could be considered ideal, it is important to note that the kinetic of release is a parameter that is also influenced by the percentage of chitosan [82]. Drugs, such as statins [83], doxycycline [84], or other antibiotics/antiseptics such as tetracyclines, have been incorporated in such devices [85,86,87].

Interestingly, adjuvant use of such drug-loaded scaffolds improved periodontal healing, emphasizing the interest in their use [78]. For instance, Chang et al. evaluated in vivo, in an experimental periodontitis murine model, the use of a chitosan-based hydrogel loaded with naringin, a natural compound with anti-inflammatory properties. They demonstrated the interesting properties of chitosan hydrogel, as its injection at the lesion site was associated with a burst release of the active compound during the acute phase of the inflammation, inducing an anti-inflammatory effect within periodontal tissues [88]. Such results might be explained by the controlled local delivery at the lesion site and the 3D characteristics of the chitosan scaffold.

Interestingly, it was also demonstrated that chitosan can potentialize the antibacterial effect of chlorhexidine [89], showing the intrinsic property of chitosan.

### 2.6. Chitosan-Based Scaffold in Dental-Pulp Regeneration

The endodontic space located in the heart of the tooth contains pulp, which is crucial for its vitality. This soft connective tissue contains collagen fibers, dental stem cells, vascularization, and innervation surrounded by dentin. This pulp-dentin complex is very reactive and, after significant aggression, the painful inflammatory reaction of the pulp–dentin complex is often hard to dissipate and leads to pulp necrosis. The conventional endodontic treatment is drastic, as all tissues are removed whatever their regenerative potentials. Recently, regenerative strategies targeting regeneration of pulp connective-tissue, dentin, radicular edification, vascularization, and innervation were developed [143]. Scaffolds represent the key element of the endodontic treatment. They are essential for delivering active molecules and for carrying competent cells within the endodontic compartment. They must have an adequate viscosity, allowing their injection in the whole root canal system, and porosity, which is crucial for cell colonization.

Recently, chitosan-based scaffolds have been developed for this purpose [144]. They are favorable, not only for pulp regeneration, but also for dentin formation because of their capacities to induce mineralization. Indeed, chitosan scaffolds containing β-tricalcium phosphate promoted a high expression of mineralization markers, such as osteopontin and alkaline phosphatase, and dentin formation by human periodontal ligament cells (HPLCs) [145]. In addition, several recent reports demonstrated that chitosan-based scaffolds promoted the proliferation, migration, and odontoblastic differentiation of dental pulp stem cells and mesenchymal stem cells both in vitro and in vivo [146,147,148,149] (Table 2). The use of chitosan as a vector for antimicrobials has also been tested with interesting outcomes [150].

## 3. Conclusions

As a positive-charged, low-cost natural polymer with good biodegradability and biocompatibility, as well as having non-toxic, muco-adhesive, hemostatic, and antimicrobial properties, chitosan is a good candidate for biomedical and biopharmaceutical research. Consequently, chitosan-based scaffolds have been widely studied and applied in tissue engineering for the last two decades. Indeed, blending chitosan with other natural or synthetic polymers and/or biomaterials could efficiently control the porosity and water retention of these biocomposite scaffolds, reduce their biodegradation rate, enhance their bioactivity and biocompatibility, and increase their mechanical properties. Importantly, as demonstrated in both in vitro and in vivo studies, most of these biocomposite scaffolds have no cytotoxicity and promote the attachment and proliferation of cells for tissue repair. In addition, the combination of bioactive molecules with these chitosan-based biocomposite scaffolds could not only promote the proliferation and differentiation of stem cells and accelerate the tissue regeneration, but also induce angiogenesis and vascularization in different animal models, which could consequently be used in human clinical trials.

## Figures and Tables

**Table 1:** In vitro and in vivo studies of chitosan-based biocomposite scaffolds in bone engineering. VEGF = vascular endothelial growth factor, TGF-β1 = transforming growth factor beta 1.

Chitosan-Based Biocomposite Scaffolds					
Polymers and/or Biomaterials		Bioactive Molecule	Models	Observations as Compared to the Properties of Chitosan Polymer Scaffolds Alone	Reference
		BMP-2	In vitro	No cytotoxicity and increased osteogenesis	[90]
			In vivo	No cytotoxicity, increased biomineralization, and increased osteogenesis	
		Recombinant human BMP-2	In vivo	Enhanced bone regeneration	[91]
			In vitro	Increased biomineralization and increased osteogenesis	[92]
			In vivo	Improved and earlier bone regeneration	[93]
			In vitro	No cytotoxicity, increased biomineralization	[94]
			In vivo	Generation of a substantial amount of bone in rat cranium	
Hydroxyapatite			In vivo	New bone tissue formation in rat	[95]
Nano hydroxyapatite			In vivo	Regeneration of segmental bone defects with cortical bone in rabbit	[96]
Nano hydroxyapatite/Nano ZrO_2_/Nano CaZrO3			In vitro	No cytotoxicity, decreased water retention and increased mechanical properties	[97]
Calcium sulfate			In vivo	Early bony consolidation	[98]
SiO_2_ + ZrO_2_			In vitro	No cytotoxicity at low concentration, decreased water retention, increased protein adsorption, biomineralization, and biodegradation	[99]
Bioactive glass + carbon nanotube			In vitro	No cytotoxicity, increased water retention, biodegradation, and mechanical properties	[100]
β-tricalcium phosphate			In vitro	No cytotoxicity at low concentration, decreased biodegradation, and increased mechanical properties	[101]
			In vivo	Increased new bone formation	[102]
γ-polyglutamic acid			In vivo	Increased new bone formation	[103]
Chondroitine sulfate + apatite		BMP-2	In vivo	Enhanced bone regeneration	[104]
Bioactive glass			In vitro	Decreased water retention, increased biomineralization, biodegradation, and mechanical properties	[105]
Bioactive glass + poly lactic-co-glycolic acid (PLGA) nanoparticles			In vitro	Decreased water retention, and increased mechanical properties	[106]
Carbon nanotube			In vitro	No cytotoxicity, increased biomineralization	[107]
Keratin nanoparticles			In vitro	No cytotoxicity, increased protein adsorption and biodegradation	[108]
Glycerophosphate			In vivo	Enhanced bone regeneration	[109]
Glycerophosphate + graphene oxide			In vitro	No cytotoxicity, increased water retention, protein adsorption, biomineralization, biodegradation, and osteogenesis	[110]
poly-ɛ-caprolactone		BMP-2	In vitro	No cytotoxicity	[111]
			In vivo	Regeneration of both the subchondral bone and the cartilage in large animal model	
Chitin + Nano ZrO_2_			In vitro	No cytotoxicity, decreased water retention and biodegradation, increased biomineralization and osteogenesis	[112]
Collagen			In vivo	Enhanced bone regeneration	[113]
		BMP-2	In vitro	No cytotoxicity, increased biomineralization and osteogenesis	[114]
			In vivo	No cytotoxicity, increased biomineralization and osteogenesis	
		BMP-7	In vivo	Accelerated regeneration of alveolar bone tissue	[115]
	PLGA/Polyethylene glycol (PEG)	VEGF	In vitro	Induced angiogenesis	[116]
			In vivo	Induced angiogenesis and vascularization in rat	
	PLGA	rhBMP-2	In vitro	Controlled growth factor release rate	[117]
			In vivo	Enhanced bone formation and fast bone regeneration in dog	
	Chondroitine sulfate + hydroxyapatite		In vitro	Secretion of higher level of receptor activator of nuclear factor kappa-B ligand (RANKL) to mediate osteoclastogenesis	[118]
	Advanced platelet rich fibrin (A-PRF)		In vitro	No cytotoxicity, increased biomineralization and mechanical properties, decreased biodegradation	[119]
Alginate	Nano SiO_2_		In vitro	No cytotoxicity, decreased water retention and mechanical properties, increased protein adsorption, biomineralization, biodegradation, and osteogenesis	[120]
	Nano-sized hydroxyapatite		In vitro	No cytotoxicity, increased biomineralization, osteogenesis, and mechanical properties	[121]
	Hydroxyapatite		In vitro	No cytotoxicity	[122]
			In vivo	Strong positive effect on bone formation in mice	
		BMP-2	In vitro	No cytotoxicity	[123]
			In vivo	Great osteogenesis and reconstruction of critical size bone defects	
Silk fibroin	Nano ZrO_2_		In vitro	No cytotoxicity, increased water retention, biomineralization, biodegradation, and mechanical properties	[124]
	Hydroxyapatite		In vitro	Increased biomineralization and osteogenesis	[125]
		TGF-β1	In vivo	Biocompatibility and extensive osteoconductivity and osteogenesis	[126]
Collagen + Poly(L-Lactide)	Nanohydroxyapatite	BMP-2	In vitro	Controlled growth factor release rate and more favorable cytocompatibility	[127]
			In vivo	Accelerated regeneration of cancellous bone defect in rabbit	
Carboxyme-thylcellulose	mesoporous wollastonite		In vitro	No cytotoxicity, decreased water retention and biodegradation, increased protein adsorption, biomineralization, and osteogenesis	[128]
Gelatin			In vivo	Increased amount of new bone formation	[129]
	Hydroxyapatite –montmorillonite		In vitro	Decreased biodegradation, increased biomineralization and mechanical properties	[130]
	Nano SiO_2_		In vitro	No cytotoxicity, decreased water retention, increased protein adsorption, biomineralization, biodegradation, and mechanical properties	[131]
	β-tricalcium phosphate		In vitro	No cytotoxicity, increased water retention, biomineralization, osteogenesis, and mechanical properties	[132]
	Hydroxyapatite+ titania		In vitro	No cytotoxicity, increased biomineralization and mechanical properties, decreased biodegradation	[133]
	Hydroxyapatite		In vitro	No cytotoxicity, increased osteogenesis and mechanical properties	[134]
Fucoidan	β-tricalcium phosphate		In vitro	No cytotoxicity, increased protein adsorption, biomineralization, osteogenesis, and mechanical properties	[135]
poly(propylene carbonate)			In vitro	No cytotoxicity, increased mechanical properties	[136]
Poly-3-hydro xybutyrate-co3-hydroxyvalerate (PHBV)	Hydroxyapatite		In vitro	No cytotoxicity, increased biomineralization, osteogenesis, and mechanical properties	[137]
Polyvinyl pyrrolidone	Bioactive glass		In vitro	No cytotoxicity, decreased biodegradation	[138]
Polypyrrole-alginate			In vitro	No cytotoxicity, increased biomineralization, decreased water retention, protein adsorption, and biodegradation	[139]
Polyvinyl alcohol or collagen	Bioactive glass		In vitro	No cytotoxicity, increased biomineralization and mechanical properties, decreased water retention and biodegradation	[140]
Polyvinyl alcohol			In vitro	No cytotoxicity, increased water retention, osteogenesis, and mechanical properties	[141]
			In vivo	Good cartilage healing in rabbit	
Polylactide + Alginate		VEGF	In vitro	Good VEGF release rate, enhanced neovascularization in bone healing and maintenance of bioactivity	[142]
			In vivo		

**Table 2:** Chitosan-based scaffold in dental-pulp regeneration.

Chitosan-Based Biocomposite Scaffolds				
Polymers and/or Biomaterials	Bioactive Molecule	Model	Observations	Reference
β-tricalcium phosphate		In vitro	Upregulated expressions of alkaline phosphatase (ALP) and osteopontin (OPN)	[145]
Collagen	BMP-7	In vivo	Release of BMP-7 gene Dental pulp stem cells (DPSC) differentiation into odontoblast-like cells in vitro and in vivo	[146]
Calcium-aluminate	1α,25-dihydroxyvitamin D3 (1α,25VD)	In vitro	Increased odontoblastic phenotype expression Cell migration	[147]
Fibrin		In vitro	Potent antibacterial effect Similar dental pulp-mesenchymal stem cells (DP-MSC) viability, fibroblast-like morphology, proliferation rate Type I/III collagen production capacity.	[148]
Silver-doped bioactive glass		In vitro	The proliferation of dental pulp cells (DPC) is not affected Decrease of inflammation Odontogenic differentiation of DPCs Inhibition of *Streptococcus mutans* and *Lactobacillus casei* growth	[149]
